# TIGAR, TIGAR, burning bright

**DOI:** 10.1186/2049-3002-2-1

**Published:** 2014-01-03

**Authors:** Pearl Lee, Karen H Vousden, Eric C Cheung

**Affiliations:** 1 Cancer Research-UK Beatson Institute, Switchback Road, Glasgow, G61 1BD, UK

**Keywords:** Cancer metabolism, p53, TIGAR, PFK-2/FBPase-2

## Abstract

Cancers cells shift their metabolism towards glycolysis in order to help them support the biosynthetic demands necessary to sustain cell proliferation and growth, adapt to stress and avoid excessive reactive oxygen species (ROS) accumulation. While the p53 tumor suppressor protein is known to inhibit cell growth by inducing apoptosis, senescence and cell cycle arrest, recent studies have found that p53 is also able to influence cell metabolism. TIGAR is a p53 target that functions as a fructose-2,6-bisphosphatase, thereby lowering glycolytic flux and promoting antioxidant functions. By protecting cells from oxidative stress, TIGAR may mediate some of the tumor suppressor activity of p53 but could also contribute to tumorigenesis. Here we discuss the activities of TIGAR described so far, and the potential consequences of TIGAR expression on normal and tumor cells.

## Review

### Introduction

Cellular metabolism is a highly regulated process through which cell growth and survival are maintained. Nutrient availability promotes the production of biosynthetic compounds for cell growth and proliferation whereas starvation halts cell proliferation in order to conserve energy and assist in cell survival [1]. Many cancer cells shift their metabolism towards glycolysis - even under aerobic conditions - in order to provide a rapid production of energy and allow for the diversion of metabolic intermediates into anabolic pathways, as well as helping in the adaptation to challenging microenvironments. This increase in aerobic glycolysis is also known as the Warburg effect [2].

Alterations in metabolic pathways have been shown to play a role in tumorigenesis, with mutations as well as changes in the expression of metabolic enzymes contributing to metabolic transformation [3]. Activated oncogenes or the loss or inhibition of tumor suppressor proteins also directly influence cancer cell metabolism and cancer cell growth [4]. Oncogenic KRas, for example, enhances the flux of glycolytic intermediates to support anabolism [5] as well as influencing the transcriptional regulation of metabolic enzymes involved in glutamine metabolism [6]. Moreover, Ras-transformed cells can stimulate macropinocytosis in order to take up extracellular proteins and provide amino acids for central carbon metabolism [7]. The transcription factor *MYC* directly activates genes involved in glucose metabolism [8,9] as well as those involved in glutamine metabolism such as glutaminase and glutamine transporters [10,11]. Hypoxia-inducible factor-1 (HIF-1), the major transcription factor involved in regulating the adaption of cells to hypoxic conditions, also regulates the expression of many glycolytic genes [12] and can be activated in cancers even under normoxic (or pseudohypoxic) conditions in response to oncogenic signaling pathways or mutations in tumor suppressor proteins [13,14]. Cancers frequently show increased PI3K-Akt growth signaling and enhanced mammalian target of rapamycin (mTOR) activity. mTOR plays a central role in cellular metabolism by regulating growth-related processes such as protein synthesis, transcription and nutrient uptake, as well as autophagy, in response to changes in cellular nutrient and energy homeostasis. Many oncogenic events converge on the regulation of mTOR, including the loss of tumor suppressors such as PTEN [15], TSC1/TSC2 and LKB1 [16].

Oncogene activation, deregulated proliferation and altered metabolic activity in cancer cells can all generate increased levels of reactive oxygen species (ROS) [3,17]. While low levels of ROS can help to promote cell proliferation, oncogenic transformation promotes the production of excessive ROS, which would become toxic if not counteracted. Therefore, many cancer cells show an increased expression of antioxidant proteins such as Nrf2 [18,19], which contribute to the survival and success of the tumor. Indeed, this dependence on antioxidants may make cancer cells more vulnerable to the inhibition of these detoxifying systems than normal cells, which do not carry such a high burden of oxidative stress [20-22].

### p53 and cancer metabolism

The p53 tumor suppressor protein functions as a transcription factor and can initiate various cellular responses, including cell cycle arrest, senescence and apoptosis [23]. However, recent studies have suggested that none of these activities are essential to protect from cancer development [24], raising the possibility that other p53 functions are important for limiting tumorigenesis. Interest has now turned to the more recently described activities of p53 in regulating metabolism and allowing cells to adapt to and survive modest or transient periods of metabolic stress [25]. These survival activities of p53 have been linked with the promotion of catabolic pathways such as fatty acid oxidation and autophagy, which may provide alternative energy sources during starvation [26]. In addition, numerous activities of p53 that assist in limiting ROS and oxidative stress through the induction of target genes such as the tumor protein p53-induced nuclear protein 1 (TP53INP1) [27], glutaminase 2 (GLS2) [28,29], manganese superoxide dismutase (MnSOD) [30] and the sestrin family of proteins [31] also contribute to cell survival. It is not clear at present how, or even whether, these activities of p53 help prevent tumor development, although an ability to limit the accumulation of potentially oncogenic damage may be an important factor.

In contrast to its survival activity, the ability of p53 to induce senescence or cell death has been associated with an ability to induce oxidative stress. Several p53-inducible pro-oxidant genes have been described, and p53 can also limit the production of nicotinamide adenine dinucleotide phosphate (NADPH), which provides the major reducing power in cells in the form of reduced glutathione, by directly inhibiting the activity of glucose-6-phosphate dehydrogenase (G6PDH) [32] and repressing the expression of malic enzymes [33]. The anti- and pro-oxidant functions of p53 seem to mirror the ability to promote either survival or death - a complexity of the p53 response that is not fully understood. Current models suggest that these opposing functions of p53 reflect different roles in response to low or transient stress (where p53 protects cells and helps them survive and repair) and high or persistent stress (where p53 drives the elimination of the damaged cell) [34]. p53 is also activated by oxidative stress, resulting in protection from or exacerbation of damage through ROS, depending on the response. More recently, oxidative stress has been shown to drive the accumulation of p53 in the mitochondrial matrix, triggering the opening of the mitochondrial permeability transition pore (PTP) through interaction with the PTP regulator cyclophilin D, leading to mitochondrial rupture and necrosis [35].

p53 therefore plays a complex but important role in the regulation of several metabolic pathways. Much like other cellular stress signals, metabolic stress can also activate p53. The activation of AMPK during low energy levels can lead to the induction of p53 activity [36] and PI3K-Akt growth signaling can inhibit p53 by activating MDM2 to promote the degradation of p53 [37]. While mTOR signaling inhibits p53 by promoting its dephosphorylation [38], a loss of the negative regulators of mTOR - and therefore, constitutive mTOR activity – can also promote p53 activity by enhancing translation [39]. Malate dehydrogenase has also been found to bind and activate p53 to mediate cell cycle arrest and apoptosis in response to glucose deprivation [40].

One important role of p53 that is beginning to emerge is its ability to help regulate the balance between glycolysis and oxidative phosphorylation. ATP and ADP can directly alter p53 activity, with ADP promoting and ATP inhibiting the ability of p53 to bind DNA [41]. p53 counteracts the elevation of glycolytic flux observed in cancer cells through inhibiting the expression of glucose transporters, GLUT1 and GLUT4 [42], as well as decreasing the levels of phosphoglycerate mutase 1 (PGAM1), the enzyme responsible for the conversion of 3-phosphoglycerate to 2-phosphoglycerate during glycolysis [43]. p53 can also promote oxidative phosphorylation through the activation of genes such as synthesis of cytochrome c oxidase 2 (SCO2) to increase mitochondrial respiration [44], as well as promote glutamine utilization through the activation of GLS2 [28,29]. Taken together, it would seem that p53 balances metabolic flux to allow for efficient energy production while blocking anabolic pathways necessary for cell growth. Indeed, loss of p53 has been suggested to be one of the mechanisms that contribute to the acquisition of the Warburg phenotype.

p53 also plays a role in preserving mitochondrial health with several activities likely to contribute to the maintenance of mitochondrial integrity. These include the induction of genes such as the ribonucleotide reductase subunit p53R2 [45-47], whose activity is required for the stability of mitochondrial DNA and the ability of p53 to contribute to the removal of damaged mitochondria [48,49]. While these results suggest that p53 helps to maintain mitochondrial quality, other studies have also demonstrated a role for p53 in the inhibition of mitophagy, an effect that would lead to increased mitochondrial dysfunction [50-52].

There have been many reviews of the role of p53 in regulating metabolic pathways, reflecting the complex interplay between p53-mediated responses that promote cell survival and those that induce cell death [53]. Here we will focus on one aspect of the p53 response: the induction of TIGAR. Of note, it has recently become clear that the expression and activity of TIGAR can be uncoupled from the p53 response and the contribution of TIGAR to cancer development may depend on the manner by which it is regulated.

### TIGAR: a fructose-2,6-bisphosphatase

TIGAR (*TP53*-induced glycolysis and apoptosis regulator) was discovered through microarray analysis of gene expression following the activation of p53 [54,55]. The human *TIGAR* gene is located on chromosome 12p13-3 and contains six coding exons and two p53 binding sites, one upstream of the first exon (BS1) and one within the first intron (BS2). Of the two sites, BS2 is much more efficient in binding p53. In the mouse genome, *Tigar* shows a similar genomic organisation but only possesses one p53 binding site, located upstream of the first exon. TIGAR is highly conserved through vertebrate species and shares similarities with the glycolytic enzyme phosphofructokinase-2/fructose-2,6-bisphosphatase (PFK-2/FBPase-2) [55].

PFK-2/FBPase-2 is a bifunctional protein containing a kinase domain within the NH_2_-terminus and a bisphosphatase domain at the COOH-terminus. These two enzymatic activities are regulated through the formation of a dimer stabilized by interactions at the kinase domain [56]. Four different genes encode the PFK-2/FBPase-2 family of enzymes, *PFKFB1* to *PFKFB4*. While their catalytic domains are highly conserved, there are notable differences between different isoforms, including tissue specificity and preferential catalytic activity [57]. Moreover, cells have been shown to co-express different PFK-2/FBPase-2 isoforms, suggesting they each have distinct functions [58]. Both the expression and activity of PFK-2/FBPase-2 can be regulated by hormones and metabolites [59,60].

Notably, TIGAR only shares similarities with the bisphosphatase domain of PFK-2/FBPase-2 [55], with clear structural similarities despite limited amino acid conservation [55,61]. Thus, TIGAR, like FBPase-2, acts to degrade intracellular fructose-2,6-bisphosphate (F-2,6-P_2_), which is a powerful allosteric activator of phosphofructokinase-1 (PFK-1). PFK-1 catalyses the conversion of fructose-6-phosphate (F-6-P) to fructose-1,6-bisphosphate (F-1,6-P_2_) and in doing so, drives glycolysis. In addition, F-2,6-P_2_ also acts as an inhibitor of fructose-1,6-bisphosphatase (FBP1) [62], which opposes the activity of PFK-1 by converting fructose-1,6-bisphosphate to fructose-6-phosphate.

**Figure 1:**
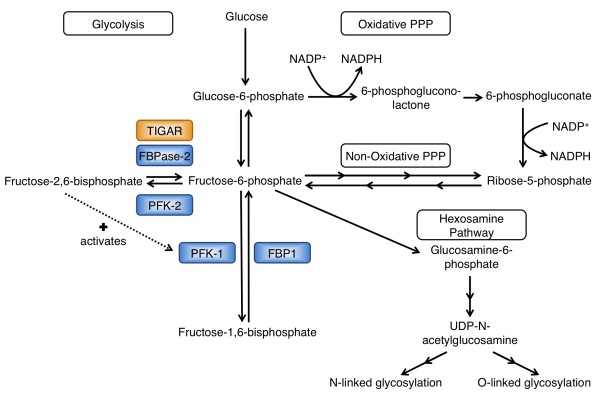
**TIGAR functions as a fructose-2,6-bisphosphatase.** Schematic of glycolysis, the pentose phosphate pathway (PPP) and the hexosamine pathway. TIGAR is predicted to promote both the PPP and hexosamine pathways by lowering the levels of fructose-2,6-bisphosphate and redirecting glycolytic intermediates.

By lowering F-2,6-P_2_ levels, TIGAR decreases the activity of PFK-1 and reduces glycolytic flux downstream of this point. Several studies have shown that depletion of TIGAR results in increased levels of F-2,6-P_2_ and increased flux through glycolysis [55,63,64], consistent with a model in which the expression of TIGAR results in a dampening, rather than a complete inhibition, of the pathway. A number of consequences of TIGAR activity can therefore be predicted, including a diversion of the glycolytic metabolites to alternative metabolic fates, such as the hexosamine pathway to support glycosylation and the oxidative or non-oxidative branches of the pentose phosphate pathway (PPP) (Figure 1). The PPP plays a key role in generating ribose-5-phosphate to be used as an intermediate in nucleotide synthesis. Furthermore, the oxidative arm of the PPP allows for the production of NADPH, which supports antioxidant function and is required for anabolic pathways such as fatty acid synthesis (Figure 1).

### The antioxidant activities of TIGAR

A dampening of glycolytic flux, either through the regulation of F-2,6-P_2_ levels [65], glycosylation of PFK-1 [66], or ROS-induced inhibition of the M2 isoform of pyruvate kinase (PKM2) [67], has been shown to lead to an elevation of NADPH and antioxidant activity, which reflects an increase in the PPP. By analogy, the FBPase-2 activity of TIGAR should result in a similar response and a number of studies have shown that the downregulation of TIGAR is associated with decreased levels of NADPH [68-70], lower levels of reduced glutathione [55,69,71] and, consequently, an increase in ROS [72].

However, the antioxidant effect of TIGAR appears to reflect more than just its FBPase-2 activity. During hypoxia, a fraction of TIGAR was found to relocalise to the mitochondria and associate with hexokinase 2 (HK2), resulting in enhanced HK2 activity, lower mitochondrial membrane potential and decreased ROS [73]. This activity displays some similarity to PFK-2/FBPase-2, where the FBPase-2 domain is able to bind and activate glucokinase (also known as hexokinase 4) [74,75]. Low oxygen availability can influence many cellular responses associated with tumor development, including angiogenesis and metastasis. In particular, hypoxia can regulate the metabolic activity of cells and induce glycolysis through the activation of HIF-1, which controls the expression of many metabolic enzymes, including *PFKFB3* and *PFKFB4*[76-78]. Notably, mutant TIGAR protein lacking FBPase-2 activity retains the ability to bind and enhance HK2 activity and the full antioxidant function of TIGAR under low-oxygen conditions depends on both HK2 binding and catalytic activity [73,79].

### TIGAR under stress

The consequences of TIGAR expression on glycolysis and ROS regulation can depend, in part, on cell type and context. For example, cytokine-dependent lymphoid cells showed a decreased growth in response to TIGAR expression, possibly in response to decreased glycolysis [55], and TIGAR was found to contribute to cell death in cardiac myocytes, an outcome that is also linked to a decrease in glycolysis [63]. However, in most cells where TIGAR functions to limit ROS, the effect of TIGAR expression was closely associated with protection against ROS-induced cell death [55,69-71,80]. More confusing is the association of TIGAR with senescence, where loss of TIGAR can induce senescence in glioblastoma cells [64] but can also inhibit this process in adult T-cell leukaemia cells [81].

**Figure 2:**
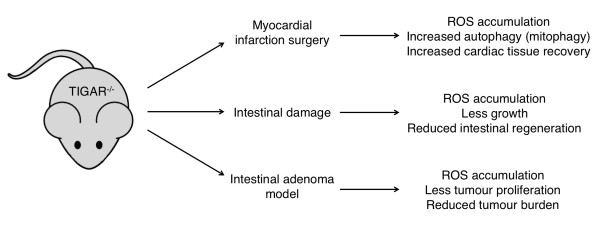
**Understanding the role of TIGAR *in vivo.*** Loss of TIGAR results in reactive oxygen species (ROS) accumulation and tissue-dependent changes in response to stress [51,79].

A clearer understanding of the physiological role of TIGAR can be provided by the analysis of the role of TIGAR *in vivo* (Figure 2)*.* Unlike many metabolic enzymes, which are essential for normal development [82,83], TIGAR deficient mice showed no profound developmental defect [79]. However, these mice have revealed a role for TIGAR in the response to various forms of stress, such as cancer and heart failure.

Cardiac myocytes are known to undergo cell death following ischaemia-reperfusion injury, where greater tissue damage occurs due to the return of oxygenated blood following an ischaemic period, resulting in inflammation and oxidative stress. Both p53 and TIGAR protein expression are induced after myocardial infarction surgery, and both have been linked to an increase in apoptosis due to a decrease in glycolysis, resulting in decreased levels of phosphocreatine (a high energy phosphate important in tissues, such as muscles, with high fluctuating energy demands) [63]. In addition, the role of p53 and TIGAR following cardiac damage was also suggested to be due to their ability to inhibit autophagy, particularly in the form of mitophagy. p53- or TIGAR-deficient animals were able to increase mitophagy after cardiac injury to reduce the number of damaged mitochondria and, hence, showed increased recovery in these tissues. In this case, the increase of ROS, due to the lack of TIGAR, functions as a signal to increase Bnip3 expression, resulting in an increase in mitophagy [51]. While a role for p53 in mediating adverse pathologies through the induction of cell death has been suggested in several diseases such as diabetes and ischaemia, protection due to a lack of TIGAR in this response is unanticipated [51,63].

More consistent with the antioxidant functions of TIGAR as protective for cell survival, as described *in vitro*, is the role of TIGAR in promoting recovery from stress-induced damage during intestinal tissue regeneration. Following ablation of the intestinal epithelium through whole body irradiation or genotoxic stress, mice deficient for TIGAR showed reduced regenerative capacity in their intestinal crypts [79]. Similarly, in a model of ulcerative colitis in the colon [84], mice that were deficient for TIGAR showed poorer recovery. As seen in cultured cells, a loss of TIGAR expression was accompanied by an increase in ROS. A lack of TIGAR compromised the ability of cells to undergo proliferation in order to regenerate the intestinal epithelium after ablation [79]. Further investigation using an *in vitro* intestinal crypt culture model [85] showed that organoids lacking TIGAR are less able to form crypt structures in a three-dimensional tissue culture model. These defects in *TIGAR*^*-/-*^ cells could be rescued following the addition of nucleosides or the antioxidant *N*-acetyl L-cysteine (NAC), suggesting that TIGAR acts to provide antioxidants and precursors for nucleic acid synthesis for intestinal growth [79].

### TIGAR in cancer

The remodelling of metabolic pathways to help the control of redox homeostasis and provide intermediates needed for cell growth is of particular importance in tumor development. The identification of TIGAR as a p53 target gene indicates some role in tumor suppression, and the antioxidant functions of TIGAR would be consistent with a role in the protective p53 response to transient or repairable stress. Indeed, while TIGAR is induced during the early stages of a p53 response, a fall in TIGAR protein levels was shown to accompany the switch to apoptosis in cells under persistent p53-activating stress [55]. These results suggest that TIGAR levels must be tightly regulated during a p53 response, and there is now growing evidence that the deregulated expression of TIGAR may contribute to cancer development.

Studies on the PFK-2/FBPase-2 family have already revealed a role for these enzymes in tumor development. All *PFKFB* mRNAs have been reported to be overexpressed in human lung cancers [86] and PFKFB3, which has predominantly kinase activity, has been suggested to promote tumorigenesis by enhancing PFK-1 activity and glycolytic flux [87]. Moreover, a recent study found a role for PFKFB3 in the proliferation of stalk endothelial cells and vessel sprouting by influencing the formation of filopodia/lamellipodia as well as cell migration. The loss of PFKFB3 in endothelial cells resulted in vascular defects *in vivo*, illustrating the importance of glycolysis in regulating vessel branching [88]. On the other hand, PFKFB4 plays an essential role in the survival of glioma stem-like cells and loss of PFKFB4 induced apoptosis in these cells [89]. Similarly in prostate cancer cells, loss of *PFKFB4* is detrimental to cell viability and resulted in a decrease in F-2,6-P_2_[65]. PFKFB4 shows predominantly bisphosphatase activity, leading to the suggestion that these cancer cells rely on PFKFB4 to dampen glycolytic flux, promote the PPP and manage ROS accumulation - very similar to the proposed action of TIGAR. However, this response to PFKFB4 expression may be more complicated than simply functioning to inhibit the PFK-1 step in glycolysis. While the inhibition of PFK-1 activity through glycosylation has been shown to promote the PPP and growth of cancer cells [66], loss of FBP1, whose activity directly opposes that of PFK-1 by converting F-1,6-P_2_ to F-6-P, has also been observed in human liver, colon, gastric and breast cancers [90,91]. Interestingly, in this context, FBP1 expression is associated not only with decreased glycolysis and enhanced flux through the TCA cycle, but also with decreased PPP flux, and thereby an increase in ROS [92]. At first glance, these results seem contradictory to the model proposed for TIGAR and PFKFB4 expression, both of which also dampen glycolysis but appear to promote the PPP. While it is difficult to compare different models and tissue types in this way, these results may reflect the functions of TIGAR that are additional to the regulation of the PFK-1/FBP1 step of glycolysis. Most clearly, the ability of TIGAR to bind to and activate HK2 [73] could profoundly influence the availability of glucose intermediates for use in pathways such as the PPP. Consistently, HK2 was found to be important in maintaining tumor proliferation in a mouse model of KRas-driven lung cancer by promoting the PPP [93].

Given the activities of TIGAR in lowering ROS and promoting anabolic pathways, and the contribution of these pathways to cancer development, it is perhaps not surprising that overexpression of TIGAR has been described in a number of tumor types. Increase in TIGAR protein expression was observed in primary colon cancer and associated metastases [79], as well as in invasive breast cancer when compared to normal tissue [94]. Glioblastoma have been found to show a high expression of TIGAR compared to normal brain tissue [71,95], and knockdown of TIGAR resulted in radiosensitisation in glioma cells through an accumulation of ROS, leading to DNA damage and cellular senescence [64]. Inhibition of transketolase-like 1, an enzyme involved in the PPP, was able to reverse the beneficial effects of TIGAR in these cells, further supporting the importance of the PPP in this response [71].

The role of TIGAR in balancing redox state in cancer cells has also been implicated in multiple myeloma cells, where inhibition of the oncoprotein MUC1-C resulted in a downregulation of TIGAR protein, lower levels of NADPH and in turn, increased ROS and cell death [69]. Moreover, in nasopharyngeal cancer cells, inhibition of c-Met, a tyrosine kinase whose overexpression has been associated with poor patient survival and metastasis [96], resulted in lower TIGAR expression, decreased NADPH and increased cell death [68].

In an intestinal adenoma model where APC is deleted in LGR5^+^ intestinal stem cells [97], mice deficient in TIGAR showed a reduction in total tumor burden and average tumor size in the small intestine compared to wild-type mice. TIGAR is also highly expressed in these adenomas when compared to the surrounding normal tissue, supporting the importance of TIGAR in proliferating tissue. A similar contribution of TIGAR to tumor progression was also observed in the colon, and importantly, the decrease in tumor burden observed in TIGAR-deficient mice correlated with a greater survival in these mice. *In vitro*, the defective growth of TIGAR-null tumor crypts could be rescued with antioxidants and nucleosides. The PPP has been shown to be of particular importance in redox homeostasis under hypoxic conditions, and TIGAR-deficient crypts were found to be more sensitive to hypoxia than wild-type crypts [79].

While the ability of TIGAR to promote cancer development might appear counterintuitive to its function in the p53 tumor suppressor pathway, it is important to note that in tumor cells overexpressing TIGAR, expression of TIGAR is uncoupled from p53 expression. Indeed, closer analysis in tumor cell lines showed that the basal expression of TIGAR is not dependent on the maintenance of wild-type p53 [79]. The ability of a p53-target protein to become oncogenic when no longer properly controlled has also been described for other mediators of the p53 survival response, such as carnitine palmitoyltransferase 1C [98]. Understanding how these genes are regulated will be critical in determining their role in cancer development.

### The regulation of TIGAR

Initial studies identified TIGAR as a p53-responsive gene [51,54,55,63,71,81]. However, p53-independent expression of TIGAR has also been seen in several human cancer cell lines [79], and its expression in human breast cancer was inversely correlated to the expression of p53 [94]. Little is known about p53-independent regulation of TIGAR, which could be transcriptional, translational, through the control of protein stability or through other post-translational modifications of the protein. Other members of the p53 family, p63 and p73, can activate promoters of several p53 target genes such as p21 and Bax [99,100], and could, therefore, also be capable of regulating TIGAR expression. While the TAp73 isoform has recently been found to be able to increase PPP activity through direct activation of G6PDH to support tumor cell proliferation [101], it is possible that the regulation of TIGAR also contributes to this response. In addition, mutant forms of p53 often display an oncogenic gain of function that can also involve modulation of tumor cell metabolism. While mutant p53s generally lose the ability to activate wild-type p53 target genes, they retain the ability to control transcription, such as the activation of genes involved in the mevalonate pathway in breast cancer cells [102]. As TIGAR expression is preserved in tumor cells that carry mutations in p53 [79], it is possible that some mutant p53s retain the ability to influence the expression of TIGAR and so help to promote tumorigenesis. Another transcription factor, SP1, has been found to regulate the basal level of TIGAR expression in liver cancer cell lines [103]. While *PFKFB3* expression can be induced by HIF-1 [76-78], TIGAR expression levels are not controlled by hypoxia. However, as discussed above, the activity of TIGAR is clearly modulated under conditions of low oxygen. Moreover, the loss of FBP1 observed in a number of human cancers and breast cancer cell lines was found to be due to promoter DNA methylation, demonstrating that epigenetic regulation also plays an important role in governing metabolism in cancer [90-92]. There is still much to be learnt about how TIGAR expression and activity are controlled under normal as well as stressed conditions.

## Conclusions

As we gain further insight into the roles of TIGAR under normal and disease conditions, we can begin to make predictions about the benefit of modulating TIGAR for therapeutic intervention*. In vivo* studies have shown that the expression of TIGAR appears to be beneficial in certain circumstances, as seen in allowing for the recovery of intestinal epithelium following damage-induced ablation, but can also be detrimental, for example in promoting cardiac damage following ischaemic stress.

The situation seems somewhat clearer in cancer development, where overexpression of TIGAR is found in several tumor types and the deletion of TIGAR corresponds to a delay in cancer development. Indeed, using conditionally expressed TIGAR alleles, TIGAR loss was beneficial subsequent to tumor establishment, providing some indication that TIGAR may be a useful therapeutic target [71,79,94]. While these effects of TIGAR loss are consistent with the observation that inhibition of other antioxidants can lead to excessive ROS and cell death in several cancer types [20-22], further investigation into TIGAR’s activity, regulation, localisation and possible post-translational modifications are required to fully understand the role of TIGAR in the control of normal and disease pathologies.

## Abbreviations

AMPK: AMP-activated protein kinase; APC: Adenomatous polyposis coli; Bnip3: BCL2/adenovirus E1B 19 kDa interacting protein 3; F-6-P: Fructose-6-phosphate; F-1,6-P2: Fructose-1,6-bisphosphate; F-2,6-P2: Fructose-2,6-bisphosphate; FBP1: Fructose-1,6-bisphosphatase; FBPase2: Fructose-2,6-bisphosphatase; G6PDH: Glucose-6-phosphate dehydrogenase; GLS2: Glutaminase 2; HIF-1: Hypoxia-inducible factor-1; HK2: Hexokinase 2; GLUT1: Glucose transporter 1; GLUT4: Glucose transporter 4; LGR5: Leucine-rich repeat containing G-protein coupled receptor 5; LKB1: Liver kinase B1; MDM2: Mouse double minute 2 homolog; MnSOD: Manganese superoxide dismutase; mTOR: Mammalian target of rapamycin; MUC1-C: Mucin 1 C-terminal subunit; NAC: *N*-acetyl L-cysteine; NADPH: Nicotinamide adenine dinucleotide phosphate; Nrf2: Nuclear factor (erythroid-derived 2)-like 2; PFK-1: Phosphofructokinase-1; PFK-2: Phosphofructokinase-2; PGAM1: Phosphoglycerate mutase 1; PI3K: Phosphatidylinositide 3-kinase; PKM2: Pyruvate kinase M2 isoform; PPP: Pentose phosphate pathway; PTEN: Phosphatase and tensin homolog; PTP: Permeability transition pore; ROS: Reactive oxygen species; SCO2: Synthesis of cytochrome c oxidase; SP1: Specificity protein 1; TCA: Tricarboxylic acid; TIGAR: *TP53*-induced glycolysis and apoptosis regulator; TP53INP1: Tumor protein p53-inducible nuclear protein 1; TSC1/TSC2: Tuberous sclerosis 1/tuberous sclerosis 2.

## Competing interests

The authors declare that they have no competing interests.

## Authors’ contributions

PL, KHV and ECC wrote the article. All authors read and approved the final manuscript.
